# The Orphan Nuclear Receptor ERRγ Regulates Hepatic CB1 Receptor-Mediated Fibroblast Growth Factor 21 Gene Expression

**DOI:** 10.1371/journal.pone.0159425

**Published:** 2016-07-25

**Authors:** Yoon Seok Jung, Ji-Min Lee, Don-Kyu Kim, Yong-Soo Lee, Ki-Sun Kim, Yong-Hoon Kim, Jina Kim, Myung-Shik Lee, In-Kyu Lee, Seong Heon Kim, Sung Jin Cho, Won-Il Jeong, Chul-Ho Lee, Robert A. Harris, Hueng-Sik Choi

**Affiliations:** 1 National Creative Research Initiatives Center for Nuclear Receptor Signals and Hormone Research Center, School of Biological Sciences and Technology, Chonnam National University, Gwangju, 61186, Republic of Korea; 2 Korea Research Institute of Bioscience and Biotechnology, Daejeon, 34141, Republic of Korea; 3 New Drug Development Center, Daegu-Gyeongbuk Medical Innovation Foundation, Daegu, 41061, Republic of Korea; 4 Severance Biomedical Science Institute and Department of Internal Medicine, Yonsei University College of Medicine, Seoul, 03722, Republic of Korea; 5 Department of Internal Medicine, School of Medicine, Kyungpook National University, Daegu, 41944, Republic of Korea; 6 Leading-edge Research Center for Drug Discovery and Development for Diabetes and Metabolic Disease, Kyungpook National University Hospital, Daegu, 41404, Republic of Korea; 7 Boryung Central Research Institute, Ansan, 15425, Republic of Korea; 8 Graduate School of Medical Science and Engineering, Korea Advanced Institute of Science and Technology, Daejeon, 34141, Republic of Korea; 9 Richard Roudebush Veterans Affairs Medical Center and the Department of Biochemistry and Molecular Biology, Indiana University School of Medicine, Indianapolis, 46202, Indiana, United States of America; Institut de Génomique Fonctionnelle de Lyon, FRANCE

## Abstract

**Background:**

Fibroblast growth factor 21 (FGF21), a stress inducible hepatokine, is synthesized in the liver and plays important roles in glucose and lipid metabolism. However, the mechanism of hepatic cannabinoid type 1 (CB1) receptor-mediated induction of FGF21 gene expression is largely unknown.

**Results:**

Activation of the hepatic CB1 receptor by arachidonyl-2’-chloroethylamide (ACEA), a CB1 receptor selective agonist, significantly increased *FGF21* gene expression. Overexpression of estrogen-related receptor (ERR) γ increased *FGF21* gene expression and secretion both in hepatocytes and mice, whereas knockdown of ERRγ decreased ACEA-mediated *FGF21* gene expression and secretion. Moreover, ERRγ, but not ERRα and ERRβ, induced *FGF21* gene promoter activity. In addition, deletion and mutation analysis of the *FGF21* promoter identified a putative ERRγ-binding motif (AGGTGC, a near-consensus response element). A chromatin immunoprecipitation assay revealed direct binding of ERRγ to the *FGF21* gene promoter. Finally, GSK5182, an ERRγ inverse agonist, significantly inhibited hepatic CB1 receptor-mediated *FGF21* gene expression and secretion.

**Conclusion:**

Based on our data, we conclude that ERRγ plays a key role in hepatic CB1 receptor-mediated induction of *FGF21* gene expression and secretion.

## Introduction

The three estrogen-related receptors (ERRs), termed α, β, and γ, belong to the NR3B subfamily of the nuclear receptor superfamily. ERRs bind to the estrogen response element as dimers or to the half-site core sequence (TNAAGGTCA) as monomers. ERR isoforms are expressed in the pancreas, heart, brain, and liver [1–3]. ERRγ plays important regulatory roles in various metabolic events. ERRs are regulated by the peripheral circadian clock in key metabolic tissues, such as muscle, white or brown adipocytes, and liver [4]. ERRγ plays an essential role in the maturation of glucose-response β-cells [5]. In brown adipose tissue, ERRγ induces Uncoupling Protein 1 (UCP1) expression and fatty acid oxidation [6]. It is also important in cancer therapy, where it is used as a marker of clinical course and in the selection of appropriate therapies [7]. ERRγ suppressed tumor growth and the proliferation of prostate cancer cells [8]. We also reported that ERRγ is involved in insulin-mediated inhibition of hepatic gluconeogenesis [9]. In addition, GSK5182 controls ERRγ-induced hepcidin gene expression and improves *Salmonella typhimurium* infection by modulating host iron homeostasis [10]. Previously, we demonstrated that hepatic ERRγ regulates the expression of gluconeogenic genes and blood glucose levels in a mouse model of type 2 diabetes and plays a key role in hepatic insulin signaling mediated by lipin1[11, 12]. Moreover, ERRγ displays endogenous ligand-independent constitutive transcriptional activity that depends on its interaction with coactivators or corepressors. PKB/Akt suppresses the transcriptional activity of ERRγ by promoting the phosphorylation of ERRγ at S179 and by eliciting translocation of ERRγ from the nucleus to the cytoplasm [9]. GSK5182, a 4-hydroxytamoxifen analog, is a selective inverse agonist of ERRγ [13]. GSK5182 inhibits ERRγ transcriptional activity increasing the interaction between ERRγ and the corepressor of SMILE [14].

The endocannabinoid system, which consists of two G protein-coupled receptors, CB1 and CB2 (cannabinoid receptor type 1 and 2), is an endogenous lipid signaling pathway. Anandamide and 2-arachidonyl glycerol (2-AG) are the two best characterized endogenous cannabinoid activators of CB1 and CB2 [15]. The CB1 receptor is expressed in the brain, vascular tissues, heart, and liver, whereas the CB2 receptor is expressed in most immune cells. 2-AG synthesis is achieved through the hydrolysis of diacylglycerol (DAG) by DAG lipases (DAGLα and DAGLβ) [16, 17]. Activation of the hepatic CB1 receptor promotes fatty acid synthesis and diet-induced obesity [18, 19]. The synthesis of 2-AG is promoted by alcohol-mediated upregulation of DAGLβ in hepatic stellate cells. 2-AG activates the CB1 receptor on adjacent hepatocytes by a paracrine mechanism [20]. Arachidonyl-2’-chloroethylamide (ACEA) is a synthetic CB1 receptor selective agonist [21], while AM251 is a CB1 receptor selective antagonist [22]. Previously, we reported that activation of the CB1 receptor inhibits insulin receptor signaling through cAMP-responsive element-binding protein 3-like 3 (CREBH)-mediated lipin1 gene expression in the liver [23]. We also reported that ERRγ regulates cytochrome P450 2E1 (CYP2E1) expression and oxidative liver injury by alcohol via the hepatic CB1 receptor [24].

Fibroblast growth factor (FGF) 21 is a member of the FGF family [25] and is a metabolic hormone secreted predominantly by hepatocytes [26]. Unlike the classical members of the FGF family, FGF21 does not have heparin-binding properties, which enables its release into the circulation [27]. Therefore, FGF21 acts through cell surface receptors composed of classic FGF receptors complexed with β-Klotho [28]. FGF21 regulates carbohydrate and lipid metabolism. For example, FGF21 increases glucose uptake via the induction of glucose transporter 1 in adipose tissue [29]. FGF21 also increases fatty acid oxidation and gluconeogenesis in the liver [30–32]. FGF21 protects against acetaminophen (APAP)-induced hepatotoxicity by increasing the peroxisome proliferator-activated receptor coactivator (PGC)-1α-mediated antioxidant capacity [33]. FGF21 expression is increased in the liver in the fasting condition by activation of the nuclear receptor peroxisome proliferator-activated receptor (PPAR) α. It is also increased in the fed condition to regulate PPARγ in adipose tissue [31, 34]. Recently, the nuclear receptor retinoic acid receptor-related orphan receptor α (RORα), retinoic acid receptor β (RARβ), and farnesoid X receptor (FXR) were shown to play a role in regulating FGF21 in the liver [35–37]. Moreover, growth hormone receptor (GHR) signaling directly stimulates *FGF21* gene transcription in the liver by janus kinase 2 (JAK2)-signal transducer and activator of transcription 5 (STAT5) [38]. However, the mechanism of hepatic cannabinoid type 1 (CB1) receptor-mediated induction of FGF21 gene expression is largely unknown.

Previously, we reported that activation of the CB1 receptor induces *CREBH* gene expression and its transcriptional activity in the liver [23], and that CREBH regulates *FGF21* promoter activity [39]. We also found that hepatic *ERRγ* expression is induced by ethanol via the activation of CB1 receptor signaling [24]. In this study, we demonstrated that the orphan nuclear receptor ERRγ is responsible for CB1 receptor-mediated *FGF21* expression. Hepatic *ERRγ* gene expression is induced by activation of the CB1 receptor, and knockdown of *ERRγ* gene expression prevents CB1 receptor-mediated *FGF21* expression. Moreover, *FGF21* gene expression and secretion are inhibited by an inverse agonist of ERRγ. Collectively, this study demonstrates that ERRγ is a novel regulator of *FGF21* gene expression and secretion.

## Materials and Methods

### Ethics Statement

Animal experiments were approved by the Chonnam National University Animal Care and Use Committee (No. CNU-IACUC-YB-2014-39).

### Chemicals

ACEA was purchased from Tocris Bioscience. GSK5182 was synthesized as described previously [13]. GSK5182 was used at a concentration of 10 μM *in vitro* and 40 mg/kg in mouse experiments.

### Animals

Male 8-week-old C57BL/6J mice (The Jackson Laboratory, Bar Harbor, ME, USA) were used for this study. Mice were maintained at 24°C on a 12:12 h light-dark cycle. CB1 receptor-knockout (CB1^−/−^) mice were kindly provided by Dr. George Kunos at the National Institute on Alcohol Abuse and Alcoholism/NIH as described previously [40, 41]. Eight-week-old male CB1^−/−^ mice were used to obtain primary hepatocytes. All animals were allowed *ad libitum* access to food and water. For the compound studies, ACEA administration (10 mg/kg, intraperitoneal injection) was performed in wild-type mice. Ad-GFP and Ad-FLAG-ERRγ were injected via the tail vein, and mice were sacrificed on day 3 after the injection. To identify the effect of ERRγ, control and recombinant shERRγ adenoviruses were injected into mice in the presence or absence of ACEA (10 mg/kg, intraperitoneal injection). GSK5182 was administrated by intraperitoneal injection (40 mg/kg). Liver tissues from chronic alcohol diet mice were used to measured FGF21 gene expression as described previously [24]. Briefly, alcohol was administered for 4weeks (chronic alcohol model) and GSK5182 was given by oral gavage administration once-daily for the last 2 weeks of alcohol feeding. The mice were monitored once-daily after experimental injection. Mice were injected with Rompon (BAYEL) and Zoletil50 (Virbac) and sacrificed by exsanguination according to the protocol of the Chonnam National University Animal Care and Use Committee (No. CNU-IACUC-YB-2014-39).

### Plasmids and DNA constructs

The FGF21-Luc (-2078bp/+129bp) construct was described previously [42]. FGF21 ERR response element mut-Luc (^-1032bp^CAAGGTGCTT^-1022bp^ to ^-1032bp^CAAAATGCTT^-1022bp^) and FGF21 ERRE mut-Luc were generated using the QuikChange II site-directed mutagenesis kit (Stratagene, La Jolla, CA, USA). ERRα, ERRβ, and ERRγ constructs were described previously [43]. All plasmids used were confirmed by complete sequence analysis.

### Recombinant adenoviruses

Ad-GFP, Ad-FLAG-ERRγ, Ad-US, and Ad-shERRγ were described previously [14]. All viruses were purified via CsCl_2_.

### Cell culture and transient transfection assays

HepG2 (human hepatoma cells) and 293T (human embryonic kidney cells) cells were obtained as described previously [44]. AML12 cells (mouse immortalized hepatocytes) were cultured in DMEM/F-12 medium (Gibco-BRL, Grand Island, NY, USA) supplemented with insulin-transferrin-selenium (Gibco-BRL), dexamethasone (40 ng/ml; Sigma, St. Louis, MO, USA), and antibiotics in a humidified atmosphere containing 5% CO_2_ at 37°C. Transient transfections were conducted using LipofectAmine 2000 reagent (Invitrogen, Carlsbad, CA, USA) in accordance with the manufacturer’s instructions. The cells were treated with 10 μM GSK5182 unless noted otherwise. After 48 h of transfection, the cells were harvested, and luciferase activity was measured and normalized to β-galactosidase activity.

### Culture of primary hepatocytes

Mouse primary hepatocytes were isolated from C57BL/6J or CB1^−/−^ mice (male, 20–22 g) by collagenase perfusion [45]. Rat primary hepatocytes were prepared from Male 8-week-old Sprague-Dawley rats (Damul Science, Daejeon, Korea) by a collagenase perfusion method, as described previously [46]. After being allowed to adhere for 12 h, cells were infected with the indicated adenoviruses for overexpression or knockdown. Hepatocytes were treated with 10 μM ACEA and 10 μM GSK5182.

### RNA isolation and analysis

Total RNA was isolated using TRIzol reagent (Invitrogen), in accordance with the manufacturer’s instructions, and real time quantitative PCR (qPCR) analysis was conducted using the following primers: ERRγ (mouse/human), 5′-AAGATCGACACATTGATTCCAGC-3′ (Forward) and 5′-CATGGTTGAACTGAATTCCCAC-3′ (Reverse); FGF21 (mouse), 5′-CTGCTGGGGGTCTACCAAG-3′ (Forward) and 5′-CTGCGCCTACCACTGTTCC-3′ (Reverse); and FGF21 (human), 5′-GGGATGTGGAGCTGGAAGTG-3′ (Forward) and 5′-TGGACCAGGAAGGACTCAC-3′ (Reverse). All data were normalized to β-actin (mouse/human) expression, which was determined using 5′-TCTGGCACCACACCTTCTAC-3′ (Forward) and 5′-TCGTAGATGGGCACAGTGTGG-3′ (Reverse) primers.

### Western blot analysis

Mouse liver tissue or cultured cells were lysed with RIPA buffer and subjected to immunoblot analysis as described previously [47]. The membranes were probed with anti-ERRγ (Cell Signaling Technology, Danvers, MA, USA; diluted 1:1000), anti-β-actin (AbFrontier, Seoul, Korea; diluted 1:5000) [24] and anti-FGF21 (Abcam, Cambridge, UK; diluted 1:1000) [48] antibodies.

### Measurement of FGF21 levels

Total FGF21 was extracted from cell culture medium or mouse serum. FGF21 was analyzed with a Quantikine FGF21 ELISA kit (R&D Systems, Minneapolis, MN, USA).

### Chromatin immunoprecipitation (ChIP) assay

Chromatin immunoprecipitation (ChIP) assays were performed according to the manufacturer’s protocol (Upstate Biotechnology, Lake Placid, NY, USA). Immunoprecipitation was performed using an anti-ERRγ antibody or IgG (as a negative control). After recovery of DNA, qPCR was performed using primers encompassing the *FGF21* promoter region. The primers used for PCR were as follows: -1.95 kb/-1.75 kb, 5′-TGGGTTCTCTGACTTGCACG-3′ (Forward) and 5′-CTACTCCCAGAGCATCTAGC-3′ (Reverse); and -0.95 kb/-0.75 kb, 5′-ACTCCTCTTACACACTGCTG-3′ (Forward) and 5′-TGGGATCTAGCTCTTGGGTC-3′ (Reverse).

### Statistical analyses

The results are presented as mean ± SD. Statistical differences in one factor between two groups were determined using the unpaired Student’s t test. Multiple-group comparisons were made using ANOVA. All analyses were performed using Prism 5 (GraphPad Software, La Jolla, CA).

## Results

### The hepatic *FGF21* mRNA level is increased by ACEA, a CB1 receptor agonist

**Fig 1:**
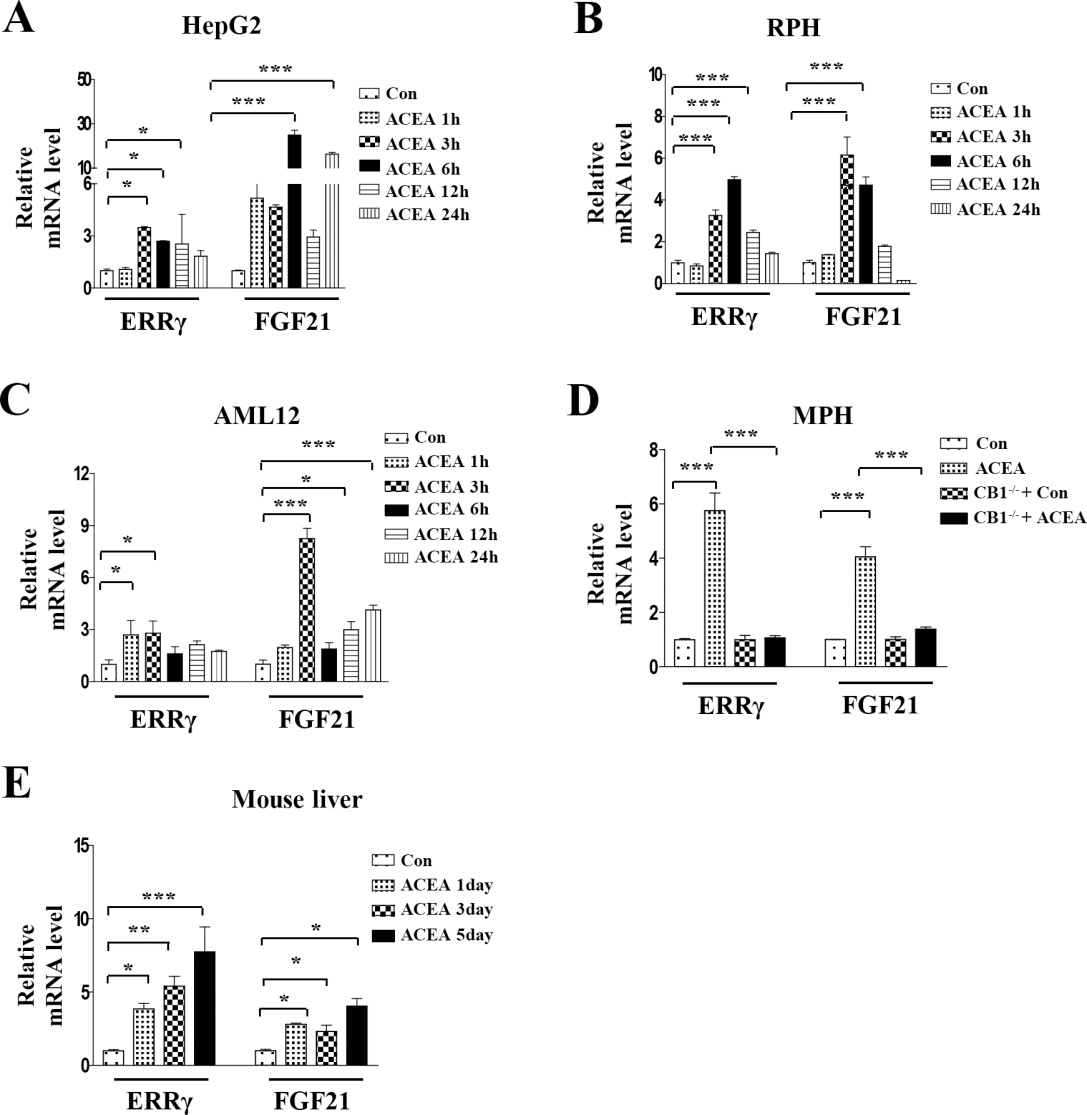
ACEA induces *FGF21* gene expression. (A–C) HepG2 cells, rat primary hepatocytes (RPH), and AML12 cells were treated with ACEA (10 μM) for the indicated time periods. (D) Wild-type or CB1^-/-^ mouse primary hepatocytes (MPH) were treated with ACEA (10 μM) for 3 h. (E) Mice were treated with ACEA (10 mg/kg) for the indicated number of days. Livers were harvested for mRNA analysis. (A–E) *FGF21* and *ERRγ* mRNA levels were measured by quantitative qPCR analysis and normalized to *actin* mRNA levels. All data are the means ± standard errors of at least three independent experiments. *p < 0.05; **p < 0.01; ***p < 0.001 by one-way ANOVA.

Previous reports demonstrated that activation of the CB1 receptor induces *CREBH* and *ERRγ* gene expression and CREBH regulates *FGF21* promoter activity [23, 24, 39]. Therefore, we hypothesized that ERRγ may regulate CB1 receptor-mediated FGF21 gene expression. We used ACEA, a CB1 receptor selective agonist, for CB1 receptor activation [21]. ACEA treatment led to significant increases in both *ERRγ* and *FGF21* mRNA levels within 1 h and reached a maximum level at 6 h in HepG2 cells and at 3 h in rat primary hepatocytes and AML12 cells (Fig 1A–1C). In mouse primary hepatocytes, ACEA also significantly increased *ERRγ* and *FGF21* mRNA levels. However, ACEA had no effect on *ERRγ* and *FGF21* mRNA levels in CB1^−/−^ mouse primary hepatocytes (Fig 1D). Together with this direct effect of ACEA on hepatocytes, we measured hepatic *ERRγ* and *FGF21* mRNA levels in ACEA-injected C57BL/6J mice. ACEA significantly increased *ERRγ* and *FGF21* mRNA levels in mouse liver in a time-dependent manner (Fig 1E). These results demonstrate that ACEA increases *ERRγ* and *FGF21* mRNA levels.

### Hepatic FGF21 is induced by ACEA

**Fig 2:**
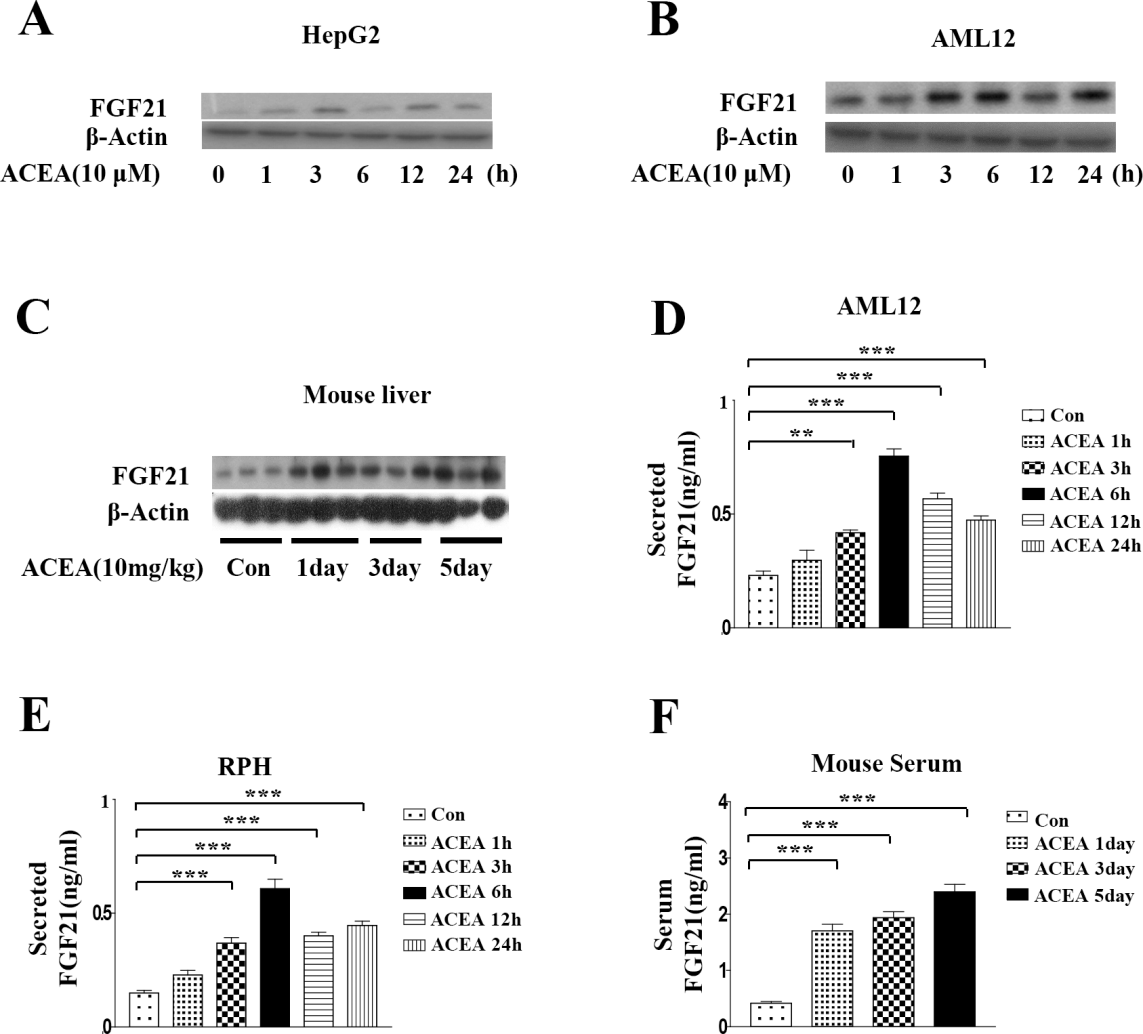
ACEA increases FGF21 protein levels. (A–C) Whole cell lysates of ACEA-treated HepG2 cells and AML12 cells and livers of ACEA-treated intact mice were harvested for western blot analysis. (D–E) AML12 cells and rat primary hepatocytes were treated with ACEA (10 μM) for the indicated time periods. Culture media were collected for FGF21 secretion analysis. (F) Mice were treated with ACEA (10 mg/kg) for the indicated number of days. Serum was obtained for FGF21 secretion analysis. All data are the means ± standard errors of at least three independent experiments. **p < 0.01; ***p < 0.001 by one-way ANOVA.

To determine whether the increases in *ERRγ* and *FGF21* mRNA levels are correlated with protein levels, western blot analysis was performed to measure FGF21 protein after ACEA treatment in HepG2 and AML12 cells. ACEA significantly increased FGF21 protein levels (Fig 2A and 2B). ACEA also induced significant increases in the FGF21 protein level in mouse liver (Fig 2C). We also examined ACEA-induced FGF21 secretion levels in AML12 cells, rat primary hepatocytes, and serum of ACEA-injected mice. ACEA treatment led to significant increases in FGF21 secretion (Fig 2D–2F). These results indicate that the CB1 receptor-specific agonist ACEA increases the protein level and secretion of FGF21.

### Overexpression of ERRγ increases hepatic FGF21 expression

**Fig 3:**
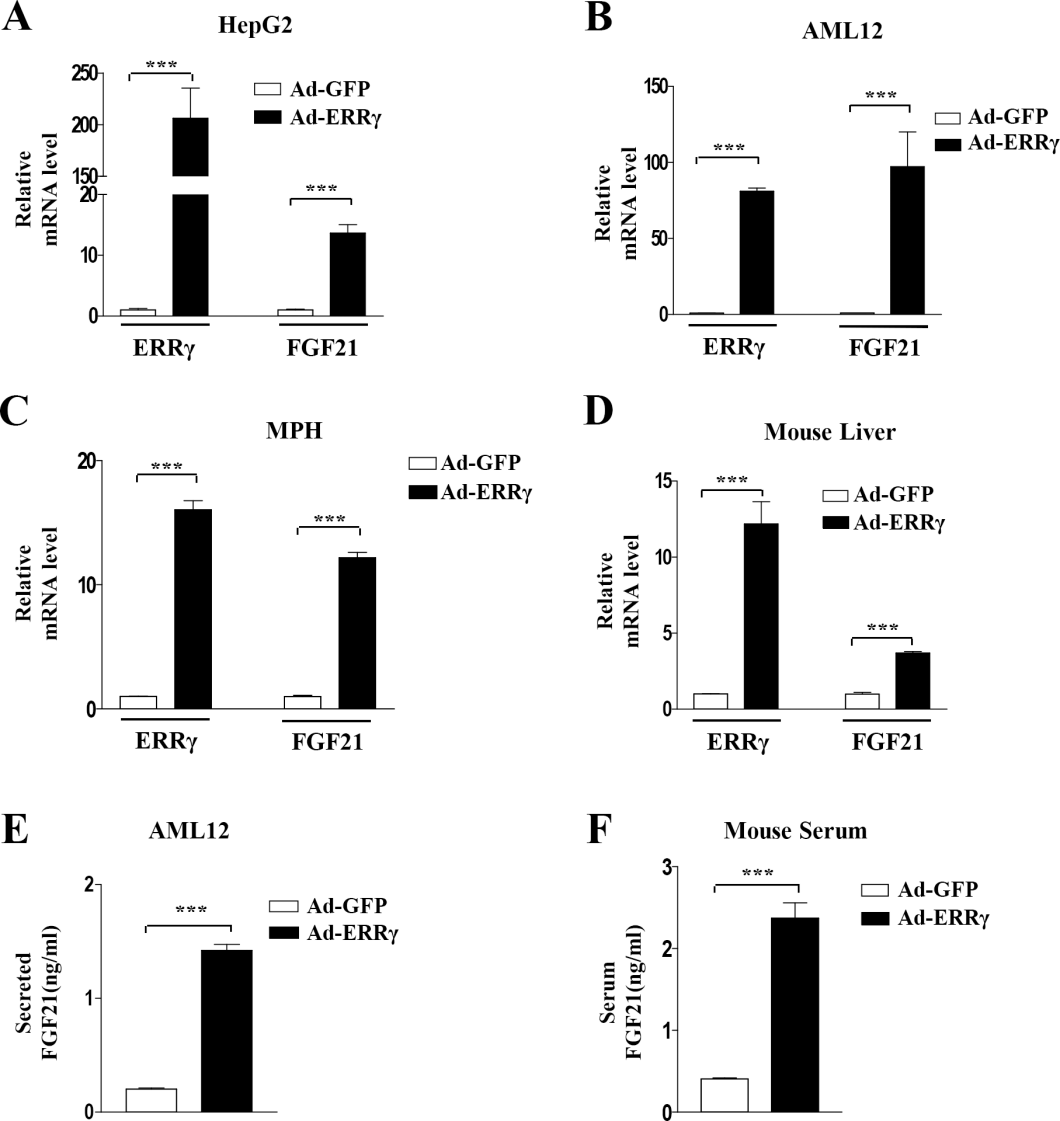
ERRγ overexpression induces *FGF21* gene expression. (A–C) HepG2 cells, AML12 cells, and mouse primary hepatocytes (MPH) were infected with Ad-GFP and Ad-ERRγ. (D) Ad-GFP or Ad-ERRγ was injected into male C57BL/6J mice via the tail vein. Mice were sacrificed at 5 days after injection. (A–D) *FGF21* and *ERRγ* mRNA levels were measured by quantitative qPCR analysis and normalized to *actin* mRNA levels. (E) Culture media of adenovirus-infected AML12 cells was obtained for FGF21 secretion analysis. (F) Ad-GFP or Ad-ERRγ was injected via the tail vein into male C57BL/6J mice. Serum from these mice was analyzed for FGF21 secretion. All data are the means ± standard errors of at least three independent experiments. ***p < 0.001 by Student’s t-test.

Our previous study demonstrated that ethanol or ACEA induces *ERRγ* gene expression through CB1 receptor signaling in the liver [24]. In this study, we found that ACEA-mediated *ERRγ* and *FGF21* expression (Figs 1 and 2). Therefore, we examined whether ERRγ regulates *FGF21* expression by overexpression of ERRγ using Ad-ERRγ in HepG2 cells, AML12 cells, and mouse primary hepatocytes. Overexpression of ERRγ markedly increased *FGF21* mRNA levels in HepG2 cells, AML12 cells, and mouse primary hepatocytes (Fig 3A–3C). Ad-ERRγ-infected mouse liver also showed significant increases in hepatic *FGF21* mRNA (Fig 3D). To verify the effect of ERRγ on FGF21 secretion, we measured FGF21 levels in both AML12 cell culture medium and mouse serum after ERRγ overexpression. Ad-ERRγ increased FGF21 levels in the culture medium and mouse serum (Fig 3E and 3F). These results suggest that ERRγ is a regulator of *FGF21* expression and secretion.

### Knockdown of ERRγ decreases ACEA-mediated FGF21 expression

**Fig 4:**
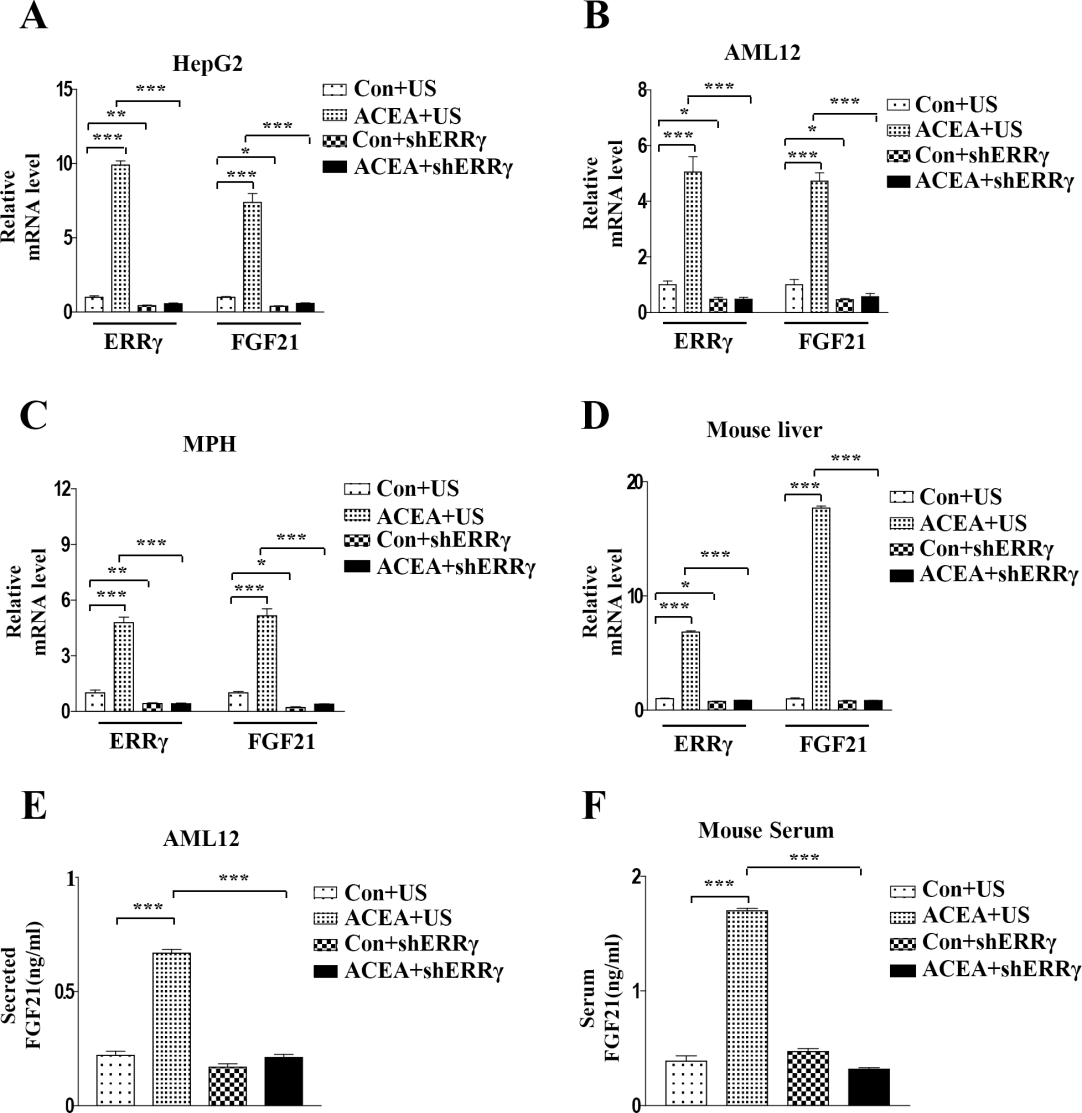
Knockdown of ERRγ decreases ACEA-mediated induction of *FGF21* gene expression. (A–C) HepG2 and AML12 cells were infected with Ad-US or Ad-shERRγ for 36 h and then treated with ACEA (10 μM) for 3 h. (D and F) Ad-US and Ad-shERRγ were injected into male C57BL/6J mice via the tail vein (n = 3–4 per group). Four days after these injections, mice were treated for 3 days with ACEA (10 mg/kg). Serum was collected for FGF21 secretion analysis. (A–D) *FGF21* and *ERRγ* mRNA levels were measured by quantitative qPCR analysis and normalized to *actin* mRNA levels. (E) AML12 cells were infected with Ad-US or Ad-shERRγ for 36 h and then treated with ACEA (10 μM) for 3 h. Culture media was collected for FGF21 secretion analysis. All data are the means ± standard errors of at least three independent experiments. *p < 0.05; ***p < 0.001 by one-way ANOVA.

To evaluate the direct effect of ERRγ on hepatic *FGF21* gene expression through the CB1 receptor, we examined ACEA-induced *FGF21* expression after knockdown of ERRγ using an adenovirus harboring ERRγ-targeting shRNA (Ad-shERRγ). The ACEA-induced *FGF21* mRNA level was dramatically reduced by knockdown of ERRγ in HepG2 cells, AML12 cells, and mouse primary hepatocytes (Fig 4A–4C). Similar to the results in hepatocytes, ACEA-induced *FGF21* expression was significantly reduced in Ad-shERRγ-infected mouse liver (Fig 4D). Furthermore, ACEA-induced FGF21 secretion in culture media of AML12 cells was significantly decreased by shERRγ. Consistent with this finding, the ACEA-induced FGF21 level in mouse serum was also significantly reduced by knockdown of ERRγ (Fig 4E and 4F). These results demonstrate that ERRγ is a key regulator of ACEA-mediated *FGF21* gene expression and secretion.

### ERRγ activates the *FGF21* gene promoter activity

**Fig 5:**
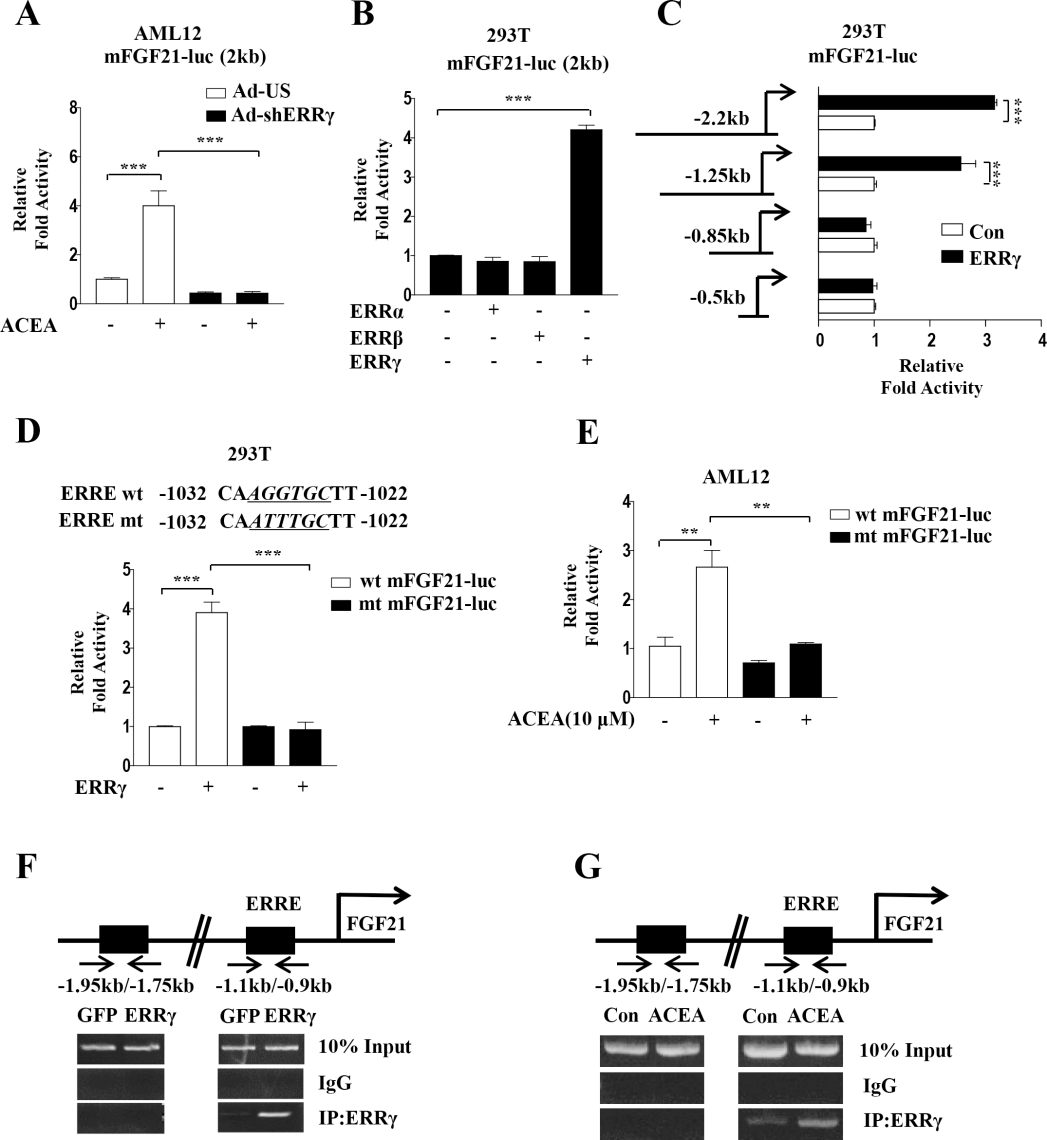
ERRγ activates mouse *FGF21* gene promoter activity. (A) AML12 cells were transfected with mFGF21-Luc and then treated with ACEA for 3 h. (B) 293T cells were transfected with mFGF21-Luc along with expression vectors for ERRα, ERRβ, and ERRγ. (C) 293T cells were co-transfected with deletion mutants of mFGF21-Luc and ERRγ. (D) 293T cells were co-transfected with mFGF21-Luc, mFGF21 ERRE mut-Luc, and ERRγ. The alignment of potential ERRE sequences in the human and mouse *FGF21* promoters is shown (top). (E) AML12 cells were transfected with mFGF21-Luc or mFGF21 ERRE mut-Luc and treated with ACEA for 3 h. (F-G) ChIP assay analysis was performed with Ad-GFP- or Ad-ERRγ-infected AML12 cells and ACEA treated AML12 cells. Cell extracts were immunoprecipitated with an anti-ERRγ antibody. Purified DNA samples were employed for PCR with primers that bind to the ERRE (-1.1 kb to -0.9 kb) and a distal site (-1.95 kb to -1.75 kb) in the *FGF21* gene promoter. All data are the means ± standard errors of at least three independent experiments. **p < 0.01; ***p < 0.001 by one-way ANOVA.

To explain the role of ERRγ in ACEA-mediated induction of *FGF21* gene expression, we investigated whether ERRγ directly induces *FGF21* gene transcription. First, we examined ACEA-induced *FGF21* promoter activity by knockdown of ERRγ using Ad-shERRγ. ACEA-induced *FGF21* promoter activity was reduced by knockdown of ERRγ in AML12 cells (Fig 5A). Next, a reporter assay with co-transfection revealed that ERRγ specifically augmented the mouse *FGF21* promoter activity, whereas ERRα and ERRβ had no effect (Fig 5B). On the other hand, serial deletion of the *FGF21* promoter showed that the region harboring -1.25 kb to -0.85 kb of the *FGF21* promoter was activated by ERRγ, and a putative ERRγ-binding motif (AGGTGC, a near-consensus response element) was identified in the *FGF21* promoter (Fig 5C). To further confirm the exact ERRγ-binding site in the *FGF21* promoter, a reporter assay was performed using wild-type and point-mutated reporter constructs of the *FGF21* promoter. ERRγ-dependent *FGF21* promoter activity was significantly decreased in the ERRE-mutated reporter construct (Fig 5D).

Next, we examined the effect of ACEA on the ERRE in the mouse *FGF21* promoter. *FGF21* promoter activity was significantly increased by ACEA. However, this ACEA-mediated *FGF21* promoter activity was decreased in ERRE-mutated reporter constructs (Fig 5E), suggesting that ACEA-dependent *FGF21* promoter activation is mediated by ERRγ. To confirm the direct binding of ERRγ to the endogenous *FGF21* promoter, ChIP assays were performed in AML12 cells. Overexpressed ERRγ and ACEA-induced endogenous ERRγ were recruited to the ERRγ consensus binding site in the *FGF21* promoter, but not to the upstream region lacking an ERRE (Fig 5F and 5G). Overall, these data suggest that activation of the hepatic CB1 receptor-induced ERRγ occupancy of the *FGF21* promoter.

### GSK5182 inhibits CB1 receptor-induced *FGF21* expression and FGF21 secretion

**Fig 6:**
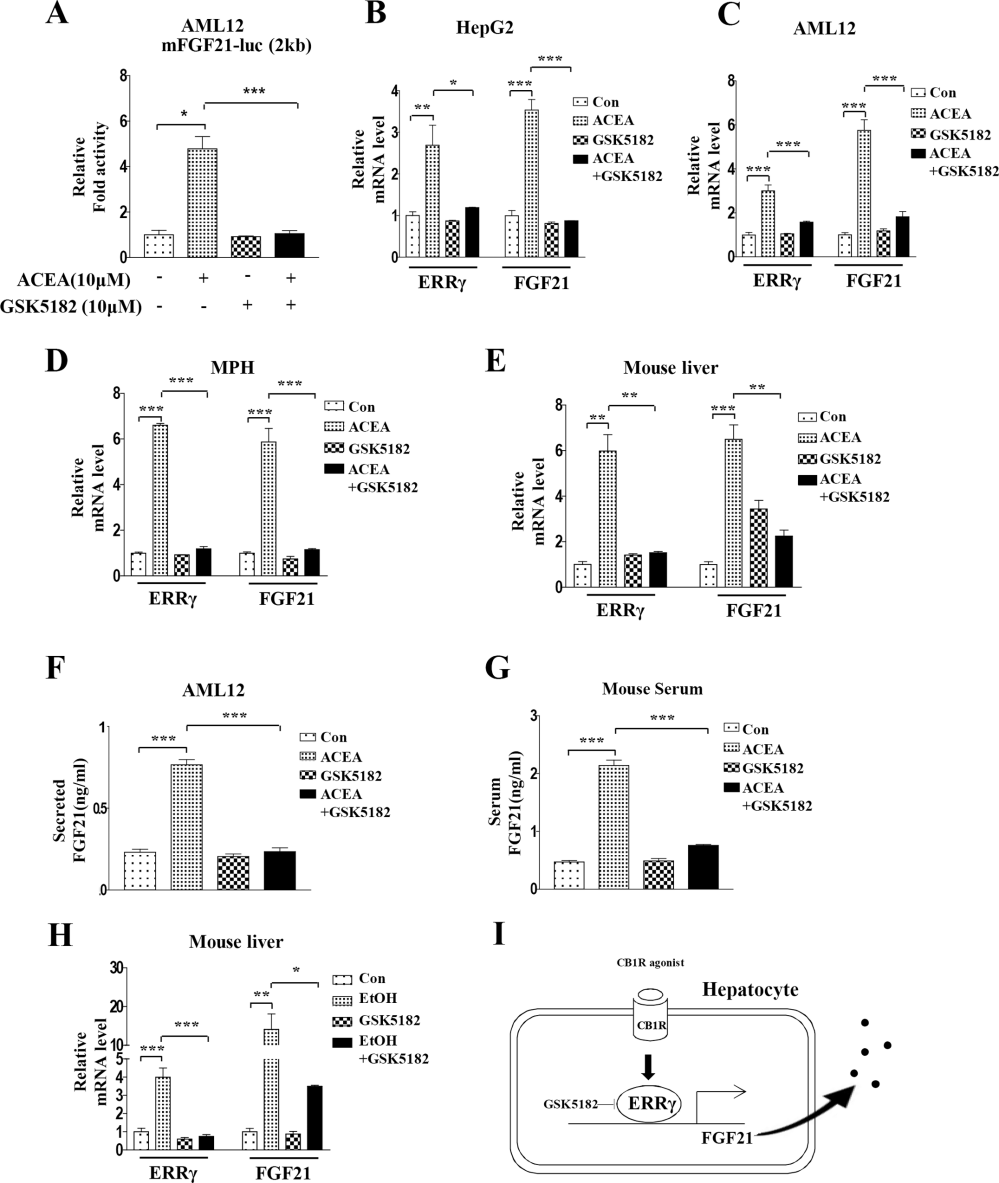
GSK5182 inhibits ACEA-mediated induction of *FGF21* gene expression. (A) AML12 cells were transfected with mFGF21-Luc and treated with ACEA (10 μM) for 3 h with or without GSK5182 (10 μM). (B–D) HepG2 cells, AML12 cells, and mouse primary hepatocytes (MPH) were treated with ACEA (10 μM) for 3 h with or without GSK5182 (10 μM). (E and G) GSK5182 (40 mg/kg) was administrated to male C57BL/6J mice (n = 3–4 per group) daily by intraperitoneal injection for 4 days. ACEA (10 mg/kg) was also given by intraperitoneal injection daily during the final 3 days. (A–D) *FGF21* and *ERRγ* mRNA levels were measured by qPCR analysis and normalized to *actin* mRNA levels. (F) AML12 cells were treated with ACEA (10 μM) for 3 h with or without GSK5182 (10 μM). Culture media was recovered for FGF21 secretion analysis. (G) Male C57BL/6J mice (n = 3) were treated with ACEA (10 mg/kg) with and without GSK5182 (40 mg/kg) daily for 3 days. Serum was analyzed for FGF21 secretion. (H) Male C57BL/6J mice (n = 5 per group) were fed an alcohol-containing diet for 4 weeks and GSK5182 (40mg/kg once daily) was given by oral gavage for the final 2 weeks of alcohol feeding. (I) Schematic diagram of ERRγ-mediated *FGF21* gene expression. GSK5182 inhibits activation of *FGF21* gene expression and FGF21 secretion mediated by increased ERRγ caused by activation of the hepatic CB1 receptor. All data are the means ± standard errors for at least three independent experiments. *p < 0.05; **p < 0.01; ***p < 0.001 by one-way ANOVA.

As an ERRγ inverse agonist, GSK5182 is used to inhibit ERRγ transactivation and its target gene expression [12, 13]. First, we examined the effect of GSK5182 on ACEA-mediated *FGF21* promoter activation. Increased *FGF21* promoter activity by ACEA was significantly decreased by GSK5182 (Fig 6A). To further clarify the direct effect of ERRγ on CB1 receptor-mediated *FGF21* expression, AML12 cells were treated with ACEA in the presence or absence of GSK5182. GSK5182 dramatically decreased *FGF21* gene expression in ACEA-treated HepG2 cells, AML12 cells, and mouse primary hepatocytes (Fig 6B–6D). In mice, the ACEA-treated group showed significant increases in *ERRγ* and *FGF21* gene expression, while GSK5182 significantly suppressed the ACEA-mediated *FGF21* mRNA level (Fig 6E). We also examined FGF21 secretion by ACEA in the presence or absence of GSK5182. GSK5182 significantly decreased ACEA-mediated FGF21 secretion in the cell culture medium (Fig 6F). In addition, the increased serum level of FGF21 induced by ACEA was also significantly reduced by GSK5182 treatment in mice (Fig 6G). Finally, we examined the effect of GSK5182 on FGF21 gene expression in a chronic alcohol-exposed C57BL6 mice model. Chronic alcohol exposure significantly increased hepatic ERRγ and FGF21 gene expression. The increase in FGF21 gene expression was decreased by GSK5182 treatment (Fig 6G). Taken together, these results indicate that the ERRγ inverse agonist GSK5182 inhibits CB1R-mediated *FGF21* expression and secretion.

## Discussion

FGF21 and ERRγ play important roles in hepatic metabolism. In this study, we revealed that ERRγ contributes to CB1 receptor-induced *FGF21* gene expression. Activation of the hepatic CB1 receptor induced *ERRγ* and *FGF21* gene expression, and knockdown of *ERRγ* gene expression prevented CB1 receptor-induced *FGF21* gene expression. In addition, ERRγ induced *FGF21* gene promoter activity by directly binding to the *FGF21* promoter. Moreover, CB1 receptor-induced *FGF21* gene expression was inhibited by the ERRγ inverse agonist GSK5182.

FGF21 is regulated by the membrane receptor GHR via the JAK2-STAT5 pathway [38]. Until now, the other membrane receptor signaling pathways regulating *FGF21* expression were largely unknown. In the present study, we demonstrated that activation of the hepatic CB1 receptor induced *FGF21* gene expression via the induction of *ERRγ* gene expression. ACEA was used to activate the CB1 receptor because ACEA is the most selective synthetic agonist of the CB1 receptor. Activation of the hepatic CB1 receptor induced FGF21 expression and secretion (Figs 1 and 2). Several nuclear receptors are involved in regulation of *FGF21* gene expression. To date, most studies have focused on transcription factors that control transcription in the -1200bp and -1000bp region of the *FGF21* gene. FGF21 is induced by PPARα in the liver during fasting by direct binding to the PPAR response element (-1093bp and -1057bp) in the *FGF21* promoter [31, 49]. *FGF21* gene expression is induced by Nur77 during fasting via direct binding of Nur77 to the NGFI-B response element (-1282bp and -1248bp) in the promoter [50]. In addition, FXR, RORα, and LXR regulate *FGF21* gene expression through direct binding to the *FGF21* promoter [35, 37, 51]. Here, we found that overexpressed ERRγ induced *FGF21* gene expression (Fig 3), and knockdown of ERRγ reduced CB1 receptor-induced FGF21 levels (Fig 4). Moreover, the present study provides an alternative mechanism by which *FGF21* promoter activity is mediated by hepatic ERRγ via the putative ERRE sequence (-1032bp and -1022bp) (Fig 5). Previously, it was reported that rosiglitazone, a PPARγ agonist, induces FGF21 in white adipose tissue, but not in the liver. All trans-retinoic acid, an endogenous ligand of RARβ, induces *FGF21* gene expression in liver tissue [36].

In this study, we demonstrated that GSK5182 inhibited hepatic CB1 receptor-induced *FGF21* gene expression via specific inhibition of the transcriptional activity of ERRγ. We also revealed that the hepatic CB1 receptor is a new membrane receptor regulating FGF21 gene expression, and that CB1 receptor-induced ERRγ is a new upstream regulator of *FGF21* gene expression. Finally, ERRγ inverse agonist GSK5182 inhibited *FGF21* gene expression in response to CB1 receptor activation. Previously, we reported that ERRγ regulates gluconeogenesis in the liver [11, 32]. ERRγ directly binds to the phosphoenolpyruvate carboxykinase (PEPCK) promoter and induces *PEPCK* gene expression. FGF21 increases hepatic gluconeogenesis through PGC-1α-mediated gluconeogenic gene expression [32]. Despite direct regulation of hepatic gluconeogenesis by ERRγ, ERRγ-induced FGF21 could be another pathway that regulates hepatic gluconeogenesis. In addition, activated CB1 receptor-induced ERRγ mediates gene expression of CYP2E1, which is a key enzyme generating alcohol-induced reactive oxygen species (ROS) in the liver [24]. In this study, hepatic *ERRγ* and *FGF21* mRNA levels were significantly increased in the chronic alcohol-exposed mice liver (Fig 6H). Other studies suggest that APAP induces hepatic ROS generation and FGF21 production. APAP-induced FGF21 protects against hepatotoxicity via inducing antioxidant gene expression [33]. Furthermore, previous study suggests that FGF21 markedly reduces alcohol preference in mice [52]. This effect requires the FGF21 co-receptor beta-klotho in the central nervous system. Previous studies suggest that the hepatic CB1 receptor contributes to obesity in a diet induced obesity (DIO) mouse model by increasing free fatty acid synthesis [18]. Paradoxically, recombinant murine FGF21 treatment reverses hepatic steatosis by increasing energy expenditure in DIO mice [53]. Our previous study revealed that ERRγ gene expression is also higher in DIO mice [54]. Therefore, we speculate that intrinsic FGF21 might be involved in CB1 receptor signaling-mediated free fatty acid synthesis. However, excessive increases in FGF21 induced by ERRγ may have a beneficial effect on hepatic steatosis. The FGF21 level is higher under stressful conditions, including starvation and high fat diet, and FGF21 improves metabolic disorder under these conditions. Therefore, our study suggests that CB1 receptor-induced ERRγ contributes to the FGF21-mediated compensatory mechanism to oppose CB1 receptor-mediated diet-induced obesity.

## Conclusions

Overall, our results reveal that ERRγ, induced via activation of the hepatic CB1 receptor, is a regulator of hepatic *FGF21* gene expression and secretion. GSK5182 inhibited CB1 receptor-mediated *FGF21* expression and secretion (Fig 6I), confirming that FGF21 is a direct target of ERRγ. In addition, chronic alcohol exposed mice showed increased FGF21 gene expression as a result of the induction of ERRγ gene expression. The identification of ERRγ as a mediator of FGF21 expression will increase current understanding of the mechanisms involved in controlling hepatic metabolism by FGF21.

## Supporting Information

S1 Fig Western blot (uncropped) for Fig 2A. (DOCX)

S2 Fig Western blot (uncropped) for Fig 2B. (DOCX)

S3 Fig Western blot (uncropped) for Fig 2C. (DOCX)

S4 Fig Electrophoresis gel of the ChIP assay (uncropped) for Fig 5F. (DOCX)

S5 Fig Electrophoresis gel of the ChIP assay (uncropped) for Fig 5G. (DOCX)

## References

[1] Giguere V, Yang N, Segui P, Evans RM. Identification of a new class of steroid hormone receptors. Nature. 1988;331(6151):91–4. 10.1038/331091a0 . 3267207

[2] Luo J, Sladek R, Carrier J, Bader JA, Richard D, Giguere V. Reduced fat mass in mice lacking orphan nuclear receptor estrogen-related receptor alpha. Molecular and cellular biology. 2003;23(22):7947–56. 14585956 10.1128/MCB.23.22.7947-7956.2003 PMC262360

[3] Razzaque MA, Masuda N, Maeda Y, Endo Y, Tsukamoto T, Osumi T. Estrogen receptor-related receptor gamma has an exceptionally broad specificity of DNA sequence recognition. Gene. 2004;340(2):275–82. 10.1016/j.gene.2004.07.010 . 15475169

[4] Yang X, Downes M, Yu RT, Bookout AL, He W, Straume M, et al Nuclear receptor expression links the circadian clock to metabolism. Cell. 2006;126(4):801–10. 10.1016/j.cell.2006.06.050 . 16923398

[5] Yoshihara E, Wei Z, Lin CS, Fang S, Ahmadian M, Kida Y, et al ERRgamma Is Required for the Metabolic Maturation of Therapeutically Functional Glucose-Responsive beta Cells. Cell metabolism. 2016;23(4):622–34. 10.1016/j.cmet.2016.03.005 27076077 PMC4832237

[6] Dixen K, Basse AL, Murholm M, Isidor MS, Hansen LH, Petersen MC, et al ERRgamma enhances UCP1 expression and fatty acid oxidation in brown adipocytes. Obesity (Silver Spring). 2013;21(3):516–24. 10.1002/oby.20067 . 23404793

[7] Ariazi EA, Clark GM, Mertz JE. Estrogen-related receptor alpha and estrogen-related receptor gamma associate with unfavorable and favorable biomarkers, respectively, in human breast cancer. Cancer Res. 2002;62(22):6510–8. . 12438245

[8] Yu S, Wang X, Ng CF, Chen S, Chan FL. ERRgamma suppresses cell proliferation and tumor growth of androgen-sensitive and androgen-insensitive prostate cancer cells and its implication as a therapeutic target for prostate cancer. Cancer Res. 2007;67(10):4904–14. 10.1158/0008-5472.CAN-06-3855 . 17510420

[9] Kim DK, Kim YH, Hynx D, Wang Y, Yang KJ, Ryu D, et al PKB/Akt phosphorylation of ERRgamma contributes to insulin-mediated inhibition of hepatic gluconeogenesis. Diabetologia. 2014;57(12):2576–85. 10.1007/s00125-014-3366-x . 25205222

[10] Kim DK, Jeong JH, Lee JM, Kim KS, Park SH, Kim YD, et al Inverse agonist of estrogen-related receptor gamma controls Salmonella typhimurium infection by modulating host iron homeostasis. Nature medicine. 2014;20(4):419–24. 10.1038/nm.3483 . 24658075

[11] Kim DK, Ryu D, Koh M, Lee MW, Lim D, Kim MJ, et al Orphan nuclear receptor estrogen-related receptor gamma (ERRgamma) is key regulator of hepatic gluconeogenesis. The Journal of biological chemistry. 2012;287(26):21628–39. 10.1074/jbc.M111.315168 22549789 PMC3381127

[12] Kim DK, Kim JR, Koh M, Kim YD, Lee JM, Chanda D, et al Estrogen-related receptor gamma (ERRgamma) is a novel transcriptional regulator of phosphatidic acid phosphatase, LIPIN1, and inhibits hepatic insulin signaling. The Journal of biological chemistry. 2011;286(44):38035–42. 10.1074/jbc.M111.250613 21911493 PMC3207427

[13] Chao EY, Collins JL, Gaillard S, Miller AB, Wang L, Orband-Miller LA, et al Structure-guided synthesis of tamoxifen analogs with improved selectivity for the orphan ERRgamma. Bioorganic & medicinal chemistry letters. 2006;16(4):821–4. 10.1016/j.bmcl.2005.11.030 . 16307879

[14] Xie YB, Park JH, Kim DK, Hwang JH, Oh S, Park SB, et al Transcriptional corepressor SMILE recruits SIRT1 to inhibit nuclear receptor estrogen receptor-related receptor gamma transactivation. The Journal of biological chemistry. 2009;284(42):28762–74. 10.1074/jbc.M109.034165 19690166 PMC2781422

[15] Sugiura T, Kobayashi Y, Oka S, Waku K. Biosynthesis and degradation of anandamide and 2-arachidonoylglycerol and their possible physiological significance. Prostaglandins, leukotrienes, and essential fatty acids. 2002;66(2–3):173–92. 10.1054/plef.2001.0356 . 12052034

[16] Bisogno T, Howell F, Williams G, Minassi A, Cascio MG, Ligresti A, et al Cloning of the first sn1-DAG lipases points to the spatial and temporal regulation of endocannabinoid signaling in the brain. The Journal of cell biology. 2003;163(3):463–8. 10.1083/jcb.200305129 14610053 PMC2173631

[17] Gao Y, Vasilyev DV, Goncalves MB, Howell FV, Hobbs C, Reisenberg M, et al Loss of retrograde endocannabinoid signaling and reduced adult neurogenesis in diacylglycerol lipase knock-out mice. The Journal of neuroscience: the official journal of the Society for Neuroscience. 2010;30(6):2017–24. 10.1523/JNEUROSCI.5693-09.2010 . 20147530 PMC6634037

[18] Osei-Hyiaman D, DePetrillo M, Pacher P, Liu J, Radaeva S, Batkai S, et al Endocannabinoid activation at hepatic CB1 receptors stimulates fatty acid synthesis and contributes to diet-induced obesity. The Journal of clinical investigation. 2005;115(5):1298–305. 10.1172/JCI23057 15864349 PMC1087161

[19] Tam J, Vemuri VK, Liu J, Batkai S, Mukhopadhyay B, Godlewski G, et al Peripheral CB1 cannabinoid receptor blockade improves cardiometabolic risk in mouse models of obesity. The Journal of clinical investigation. 2010;120(8):2953–66. 10.1172/JCI42551 20664173 PMC2912197

[20] Jeong WI, Osei-Hyiaman D, Park O, Liu J, Batkai S, Mukhopadhyay P, et al Paracrine activation of hepatic CB1 receptors by stellate cell-derived endocannabinoids mediates alcoholic fatty liver. Cell metabolism. 2008;7(3):227–35. 10.1016/j.cmet.2007.12.007 . 18316028

[21] Hillard CJ, Manna S, Greenberg MJ, DiCamelli R, Ross RA, Stevenson LA, et al Synthesis and characterization of potent and selective agonists of the neuronal cannabinoid receptor (CB1). J Pharmacol Exp Ther. 1999;289(3):1427–33. . 10336536

[22] Lan R, Liu Q, Fan P, Lin S, Fernando SR, McCallion D, et al Structure-activity relationships of pyrazole derivatives as cannabinoid receptor antagonists. J Med Chem. 1999;42(4):769–76. 10.1021/jm980363y . 10052983

[23] Chanda D, Kim YH, Kim DK, Lee MW, Lee SY, Park TS, et al Activation of cannabinoid receptor type 1 (Cb1r) disrupts hepatic insulin receptor signaling via cyclic AMP-response element-binding protein H (Crebh)-mediated induction of Lipin1 gene. The Journal of biological chemistry. 2012;287(45):38041–9. 10.1074/jbc.M112.377978 22989885 PMC3488074

[24] Kim DK, Kim YH, Jang HH, Park J, Kim JR, Koh M, et al Estrogen-related receptor gamma controls hepatic CB1 receptor-mediated CYP2E1 expression and oxidative liver injury by alcohol. Gut. 2013;62(7):1044–54. 10.1136/gutjnl-2012-303347 23023167 PMC3812689

[25] Nishimura T, Nakatake Y, Konishi M, Itoh N. Identification of a novel FGF, FGF-21, preferentially expressed in the liver. Biochimica et biophysica acta. 2000;1492(1):203–6. . 10858549 10.1016/s0167-4781(00)00067-1

[26] Itoh N, Ornitz DM. Fibroblast growth factors: from molecular evolution to roles in development, metabolism and disease. Journal of biochemistry. 2011;149(2):121–30. 10.1093/jb/mvq121 20940169 PMC3106964

[27] Kliewer SA, Mangelsdorf DJ. Fibroblast growth factor 21: from pharmacology to physiology. The American journal of clinical nutrition. 2010;91(1):254S–7S. 10.3945/ajcn.2009.28449B 19906798 PMC2793111

[28] Kharitonenkov A, Dunbar JD, Bina HA, Bright S, Moyers JS, Zhang C, et al FGF-21/FGF-21 receptor interaction and activation is determined by betaKlotho. Journal of cellular physiology. 2008;215(1):1–7. 10.1002/jcp.21357 . 18064602

[29] Kharitonenkov A, Shiyanova TL, Koester A, Ford AM, Micanovic R, Galbreath EJ, et al FGF-21 as a novel metabolic regulator. The Journal of clinical investigation. 2005;115(6):1627–35. 10.1172/JCI23606 15902306 PMC1088017

[30] Coskun T, Bina HA, Schneider MA, Dunbar JD, Hu CC, Chen Y, et al Fibroblast growth factor 21 corrects obesity in mice. Endocrinology. 2008;149(12):6018–27. 10.1210/en.2008-0816 . 18687777

[31] Inagaki T, Dutchak P, Zhao G, Ding X, Gautron L, Parameswara V, et al Endocrine regulation of the fasting response by PPARalpha-mediated induction of fibroblast growth factor 21. Cell metabolism. 2007;5(6):415–25. 10.1016/j.cmet.2007.05.003 . 17550777

[32] Potthoff MJ, Inagaki T, Satapati S, Ding X, He T, Goetz R, et al FGF21 induces PGC-1alpha and regulates carbohydrate and fatty acid metabolism during the adaptive starvation response. Proceedings of the National Academy of Sciences of the United States of America. 2009;106(26):10853–8. 10.1073/pnas.0904187106 19541642 PMC2705613

[33] Ye D, Wang Y, Li H, Jia W, Man K, Lo CM, et al Fibroblast growth factor 21 protects against acetaminophen-induced hepatotoxicity by potentiating peroxisome proliferator-activated receptor coactivator protein-1alpha-mediated antioxidant capacity in mice. Hepatology. 2014;60(3):977–89. 10.1002/hep.27060 . 24590984

[34] Dutchak PA, Katafuchi T, Bookout AL, Choi JH, Yu RT, Mangelsdorf DJ, et al Fibroblast growth factor-21 regulates PPARgamma activity and the antidiabetic actions of thiazolidinediones. Cell. 2012;148(3):556–67. 10.1016/j.cell.2011.11.062 22304921 PMC3273727

[35] Wang Y, Solt LA, Burris TP. Regulation of FGF21 expression and secretion by retinoic acid receptor-related orphan receptor alpha. The Journal of biological chemistry. 2010;285(21):15668–73. 10.1074/jbc.M110.102160 20332535 PMC2871432

[36] Li Y, Wong K, Walsh K, Gao B, Zang M. Retinoic acid receptor beta stimulates hepatic induction of fibroblast growth factor 21 to promote fatty acid oxidation and control whole-body energy homeostasis in mice. The Journal of biological chemistry. 2013;288(15):10490–504. 10.1074/jbc.M112.429852 23430257 PMC3624431

[37] Cyphert HA, Ge X, Kohan AB, Salati LM, Zhang Y, Hillgartner FB. Activation of the farnesoid X receptor induces hepatic expression and secretion of fibroblast growth factor 21. The Journal of biological chemistry. 2012;287(30):25123–38. 10.1074/jbc.M112.375907 22661717 PMC3408207

[38] Yu J, Zhao L, Wang A, Eleswarapu S, Ge X, Chen D, et al Growth hormone stimulates transcription of the fibroblast growth factor 21 gene in the liver through the signal transducer and activator of transcription 5. Endocrinology. 2012;153(2):750–8. 10.1210/en.2011-1591 . 22166977

[39] Kim H, Mendez R, Zheng Z, Chang L, Cai J, Zhang R, et al Liver-enriched transcription factor CREBH interacts with peroxisome proliferator-activated receptor alpha to regulate metabolic hormone FGF21. Endocrinology. 2014;155(3):769–82. 10.1210/en.2013-1490 24424044 PMC3929740

[40] Jarai Z, Wagner JA, Varga K, Lake KD, Compton DR, Martin BR, et al Cannabinoid-induced mesenteric vasodilation through an endothelial site distinct from CB1 or CB2 receptors. Proceedings of the National Academy of Sciences of the United States of America. 1999;96(24):14136–41. 10570211 10.1073/pnas.96.24.14136 PMC24203

[41] Wang L, Liu J, Harvey-White J, Zimmer A, Kunos G. Endocannabinoid signaling via cannabinoid receptor 1 is involved in ethanol preference and its age-dependent decline in mice. Proceedings of the National Academy of Sciences of the United States of America. 2003;100(3):1393–8. 10.1073/pnas.0336351100 12538878 PMC298783

[42] Kim KH, Jeong YT, Oh H, Kim SH, Cho JM, Kim YN, et al Autophagy deficiency leads to protection from obesity and insulin resistance by inducing Fgf21 as a mitokine. Nature medicine. 2013;19(1):83–92. 10.1038/nm.3014 . 23202295

[43] Sanyal S, Kim JY, Kim HJ, Takeda J, Lee YK, Moore DD, et al Differential regulation of the orphan nuclear receptor small heterodimer partner (SHP) gene promoter by orphan nuclear receptor ERR isoforms. The Journal of biological chemistry. 2002;277(3):1739–48. 10.1074/jbc.M106140200 . 11705994

[44] Ryu D, Oh KJ, Jo HY, Hedrick S, Kim YN, Hwang YJ, et al TORC2 regulates hepatic insulin signaling via a mammalian phosphatidic acid phosphatase, LIPIN1. Cell metabolism. 2009;9(3):240–51. 10.1016/j.cmet.2009.01.007 . 19254569

[45] Koo SH, Satoh H, Herzig S, Lee CH, Hedrick S, Kulkarni R, et al PGC-1 promotes insulin resistance in liver through PPAR-alpha-dependent induction of TRB-3. Nature medicine. 2004;10(5):530–4. 10.1038/nm1044 . 15107844

[46] Kim YD, Kim YH, Cho YM, Kim DK, Ahn SW, Lee JM, et al Metformin ameliorates IL-6-induced hepatic insulin resistance via induction of orphan nuclear receptor small heterodimer partner (SHP) in mouse models. Diabetologia. 2012;55(5):1482–94. 10.1007/s00125-012-2494-4 . 22349108

[47] Lee YS, Kim DK, Kim YD, Park KC, Shong M, Seong HA, et al Orphan nuclear receptor SHP interacts with and represses hepatocyte nuclear factor-6 (HNF-6) transactivation. The Biochemical journal. 2008;413(3):559–69. 10.1042/BJ20071637 . 18459945

[48] Loeffler I, Hopfer U, Koczan D, Wolf G. Type VIII collagen modulates TGF-beta1-induced proliferation of mesangial cells. J Am Soc Nephrol. 2011;22(4):649–63. 10.1681/ASN.2010010098 21372207 PMC3065221

[49] Fisher FM, Chui PC, Antonellis PJ, Bina HA, Kharitonenkov A, Flier JS, et al Obesity is a fibroblast growth factor 21 (FGF21)-resistant state. Diabetes. 2010;59(11):2781–9. 10.2337/db10-0193 20682689 PMC2963536

[50] Min AK, Bae KH, Jung YA, Choi YK, Kim MJ, Kim JH, et al Orphan nuclear receptor Nur77 mediates fasting-induced hepatic fibroblast growth factor 21 expression. Endocrinology. 2014;155(8):2924–31. 10.1210/en.2013-1758 . 24885573

[51] Uebanso T, Taketani Y, Yamamoto H, Amo K, Tanaka S, Arai H, et al Liver X receptor negatively regulates fibroblast growth factor 21 in the fatty liver induced by cholesterol-enriched diet. The Journal of nutritional biochemistry. 2012;23(7):785–90. 10.1016/j.jnutbio.2011.03.023 . 21889884

[52] Talukdar S, Owen BM, Song P, Hernandez G, Zhang Y, Zhou Y, et al FGF21 Regulates Sweet and Alcohol Preference. Cell metabolism. 2016;23(2):344–9. 10.1016/j.cmet.2015.12.008 26724861 PMC4749404

[53] Xu J, Lloyd DJ, Hale C, Stanislaus S, Chen M, Sivits G, et al Fibroblast growth factor 21 reverses hepatic steatosis, increases energy expenditure, and improves insulin sensitivity in diet-induced obese mice. Diabetes. 2009;58(1):250–9. 10.2337/db08-0392 18840786 PMC2606881

[54] Kim DK, Gang GT, Ryu D, Koh M, Kim YN, Kim SS, et al Inverse agonist of nuclear receptor ERRgamma mediates antidiabetic effect through inhibition of hepatic gluconeogenesis. Diabetes. 2013;62(9):3093–102. 10.2337/db12-0946 23775767 PMC3749343

