# Supplementary material for: The Orphan Nuclear Receptor ERRγ Regulates Hepatic CB1 Receptor-Mediated Fibroblast Growth Factor 21 Gene Expression
Source: PLoS One. 2016 Jul 25;11(7):e0159425. doi: 10.1371/journal.pone.0159425 (PMC4959684; doi:10.1371/journal.pone.0159425)
Supplement: S2 Fig — (DOCX) [file pone.0159425.s002.docx]

Supporting Information

FGF21

**MW**

**Ladder**

**ACEA**

**0h**

**ACEA**

**1h**

**ACEA**

**3h**

**ACEA**

**6h**

**ACEA**

**12h**

**ACEA**

**24h**


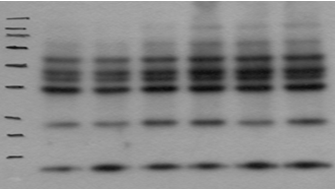


**70**

**55**

**43**

**34**

**25**

**17**

**kDa**

**MW**

**Ladder**

**ACEA**

**0h**

**ACEA**

**1h**

**ACEA**

**3h**

**ACEA**

**6h**

**ACEA**

**12h**

**ACEA**

**24h**

Beta Actin


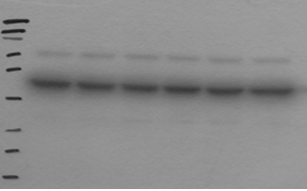


**70**

**55**

**43**

**34**

**25**

**17**

**kDa**

S2 Fig. Western blot (uncopped) for Fig 2B.
