# Supplementary material for: The Orphan Nuclear Receptor ERRγ Regulates Hepatic CB1 Receptor-Mediated Fibroblast Growth Factor 21 Gene Expression
Source: PLoS One. 2016 Jul 25;11(7):e0159425. doi: 10.1371/journal.pone.0159425 (PMC4959684; doi:10.1371/journal.pone.0159425)
Supplement: S3 Fig — (DOCX) [file pone.0159425.s003.docx]

Supporting Information

FGF21

**MW**

**Ladder**

**Con**

**ACEA**

**Day 1**

**ACEA**

**Day 3**

**ACEA**

**Day 5**


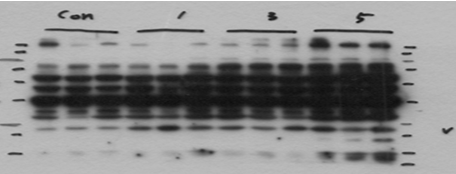


**70**

**55**

**43**

**34**

**25**

**17**

**kDa**

Beta Actin

**MW**

**Ladder**

**Con**

**ACEA**

**Day 1**

**ACEA**

**Day 3**

**ACEA**

**Day 5**


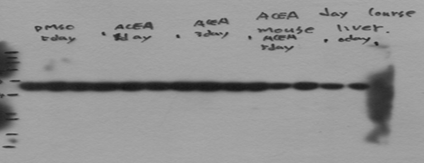


**70**

**55**

**43**

**34**

**25**

**17**

**kDa**

S3 Fig. Western blot (uncopped) for Fig 2C.
