# Supplementary figures and images for: The Orphan Nuclear Receptor ERRγ Regulates Hepatic CB1 Receptor-Mediated Fibroblast Growth Factor 21 Gene Expression
Source: PLoS One. 2016 Jul 25;11(7):e0159425. doi: 10.1371/journal.pone.0159425 (PMC4959684; doi:10.1371/journal.pone.0159425)

Supporting Information


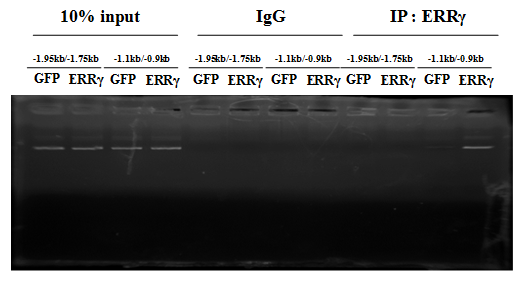


S4 Fig. Electrophoresis gel of the ChIP assay (uncropped) for Fig 5F.

Supplement: S4 Fig — (DOCX) [file pone.0159425.s004.docx]
