# Supplementary material for: The Orphan Nuclear Receptor ERRγ Regulates Hepatic CB1 Receptor-Mediated Fibroblast Growth Factor 21 Gene Expression
Source: PLoS One. 2016 Jul 25;11(7):e0159425. doi: 10.1371/journal.pone.0159425 (PMC4959684; doi:10.1371/journal.pone.0159425)
Supplement: S5 Fig — (DOCX) [file pone.0159425.s005.docx]

Supporting Information

**10% input**

**IgG**

**IP: ERRγ**

**-1.95kb/-1.75kb**

**-1.95kb/-1.75kb**

**-1.95kb/-1.75kb**

**-1.1kb/-0.9kb**

**-1.1kb/-0.9kb**

**-1.1kb/-0.9kb**

**GFP**

**ERRγ**

**GFP**

**ERRγ**

**GFP**

**ERRγ**

**GFP**

**ERRγ**

**GFP**

**ERRγ**

**GFP**

**ERRγ**


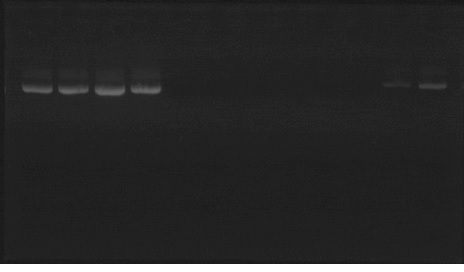


S5 Fig. Electrophoresis gel of the ChIP assay (uncropped) for Fig 5G.
